# Accelerometer-based predictive models of fall risk in older women: a pilot study

**DOI:** 10.1038/s41746-018-0033-5

**Published:** 2018-07-11

**Authors:** Andrew Hua, Zachary Quicksall, Chongzhi Di, Robert Motl, Andrea Z. LaCroix, Bruce Schatz, David M. Buchner

**Affiliations:** 1 University of Illinois at Urbana-Champaign, Urbana, IL USA; 2 Carl R. Woese Institute for Genomic Biology, Urbana, IL USA; 3 Fred Hutchinson Cancer Research Center, Seattle, WA USA; 4 University of Alabama at Birmingham, Birmingham, AL USA; 5 University of California at San Diego, San Diego, USA

**Keywords:** Geriatrics, Risk factors

## Abstract

Current clinical methods of screening older adults for fall risk have difficulties. We analyzed data on 67 women (mean age = 77.5 years) who participated in the Objective Physical Activity and Cardiovascular Health (OPACH) study within the Women’s Health Initiative and in an accelerometer calibration substudy. Participants completed the short physical performance battery (SPPB), questions about falls in the past year, and a timed 400-m walk while wearing a hip triaxial accelerometer (30 Hz). Women with SPPB ≤ 9 and 1+reported falls (*n* = 19) were grouped as high fall risk; women with SPPB = 10–12 and 0 reported falls (*n* = 48) were grouped as low fall risk. Random Forests were trained to classify women into these groups, based upon traditional measures of gait and/or signal-based features extracted from accelerometer data. Eleven models investigated combined feature effects on classification accuracy, using 10-fold cross-validation. The models had an average 73.7% accuracy, 81.1% precision, and 0.706 AUC. The best performing model including triaxial data, cross-correlations, and traditional measures of gait had 78.9% accuracy, 84.4% precision, and 0.846 AUC. Mediolateral signal-based measures—coefficient of variance, cross-correlation with anteroposterior accelerations, and mean acceleration—ranked as the top 3 features. The classification accuracy is promising, given research on probabilistic models of falls indicates accuracies ≥80% are challenging to achieve. The results suggest accelerometer-based measures captured during walking are potentially useful in screening older women for fall risk. We are applying algorithms developed in this paper on an OPACH dataset of 5000 women with a 1-year prospective falls log and week-long, free-living accelerometer data.

## Wearables: Hip-worn accelerometers predict fall risk among the elderly

A hip-worn device that measures walking motion can help identify which older women are at heightened risk for falling. Andrew Hua, from the University of Illinois at Urbana-Champaign, USA, and colleagues put 67 elderly women through a series of tests to assess their lower extremity function. They also asked the study participants about fall histories in the past year and strapped a triaxial accelerometer to the women’s hips while they completed a 400-meter walking test. Analyses showed that the accelerometry data, when fed into a machine-learning algorithm, were predictive of physical ability and fall risk. Based on these results, the researchers are validating the algorithm in a larger study of 5000 women who wore hip accelerometers for a full week and reported falls prospectively for one-year.

## Introduction

Falls are the most common cause of serious injuries in older adults. During 2014, approximately 2.8 million adults were treated for fall-related injuries in emergency departments, and about 27,000 older adults died because of falls or fall-related injuries.^1^ Accordingly, the U.S. Preventive Services Task Force recommends screening older adults for fall risk and implementing prevention strategies in high-risk adults, such as exercise programs.^2^

There are several methods of screening for fall risk. For example, the Centers for Disease Control and Prevention (CDC) has developed the *STEADI* toolkit, which includes a screening approach that combines questions about falls and functional limitations with simple physical performance tests such as the Timed Up & Go (TUG).^3^ The short physical performance battery (SPPB) assesses fall risk by measuring balance, gait, and muscular strength.^4,5^ Overall, the sensitivity and specificity of existing screening methods is modest. In one review of 38 different screening tools, there were only four methods with high specificity (over 90%) but all of them had mediocre sensitivity (50–60%).^6^ While more comprehensive assessments of fall risk have been developed, their feasibility for mass screenings is questionable. For example, Lord et al.^7^ have developed a comprehensive fall risk assessment tool which measures physiologic capacity in each organ system related to falls, but the short version of this tool requires equipment that is not readily available, 10–15 min for administration, and a trained assessor. Existing methods of screening for fall risk typically involve assessments in a clinic or laboratory, and therefore may not reflect fall risk during everyday life activity (i.e., real-world monitoring).

One potential approach for screening fall risk that may better reflect fall risk during daily life is use of wearable devices during walking tasks to characterize gait and a person’s pattern of walking during the day. While a variety of devices exist, the use of triaxial accelerometers has several desirable characteristics for screening purposes. Accelerometers are becoming more affordable and available in consumer devices such as smartphones, which are nearly ubiquitous.^8^ When motion sensors sample at 30 Hz or greater, raw data can provide precise measures of gait such as measures of gait variability among gait cycles during walking tasks.^9,10^ While many other devices can be used for brief clinical gait assessments, accelerometers offer the option of basing fall risk assessments on data collected during actual activities of daily living, including frequent longitudinal measurements from worn or carried devices.

The utility of accelerometers assumes that fall risk is consistently correlated with characteristics of body movement and gait, and that these characteristics can be accurately detected using sensors measuring body motions. Instability during movement and walking is a primary cause of actual falls, as emphasized in studies analyzing senior falls in nursing homes.^11^ Several studies have demonstrated the potential of using raw data from wearable devices to predict fall risk by identifying gait related risk factors. Some use multiple sensors across the body (head, torso, pressure insoles, etc.).^12–16^ Other studies use specialty sensors developed in the lab (not commercially available requiring hardware development to collect data) which limits applicability.^17,18^ Our study, in contrast, uses a single sensor, placed at the hip.

One concern with using a single sensor to assess fall risk is that fall risk prediction from single sensors may have limited accuracy. We are aware of one previous study that used a single pelvis accelerometer that attained lower rates of accuracy = 57%, sensitivity = 43%, and specificity = 65%.^19^ However, use of a single sensor at the hip may be feasible, as if a single sensor is to be used to predict fall risk during walking, the hip is the preferred location.^19^ The hip is responsible for connecting the lower kinetic chain (legs and feet) to the areas responsible for maintaining balance (core and head). Older adults with high fall risk exhibit reduced harmonic ratios of acceleration patterns in the pelvis.^16^ A single hip mounted accelerometer is best suited to capture these accelerations compared to L3 that is commonly used in fall risk assessment and gait analysis. Moving the accelerometer further from the midline allows for collecting small mediolateral and anteroposterior accelerations that may not be detected at the midline due to greater displacement at the lateral aspect of the hip compared to the small of the back.

In developing methods to identify accelerometer-based risk factor assessments of fall risk, our study uses the concept of the *STEADI* toolkit for classifying fall risk. Fall risk was classified using physical performance and falls history, rather than only a faller/non-faller status.^3^ This approach is distinct from recent studies which use machine learning techniques to develop models using accelerometer data to predict fall risk based on physical function and falls history.

The purpose of this pilot study is to develop a machine learning algorithm for classifying older women into high versus low fall risk categories, based upon raw data from hip-worn accelerometers collected at 30 Hz during one bout of walking (from a 400-m walk test). The study also seeks to explore which features of gait derived from the *x* (anteroposterior), *y* (vertical), and *z* (mediolateral) axes of the accelerometer are potentially useful for assessing fall risk. The results of this pilot study are informing the methods of an ongoing larger prospective study of accelerometer-based fall prediction during activities of daily living, leveraging the full dataset of the Objective Physical Activity and Cardiovascular Health (OPACH) study within the Women’s Health Initiative. Ultimately, the results may be used to develop a self-administered fall risk assessment for home-based delivery using a single hip sensor, such as an embedded sensor within an inexpensive smartphone as carried during daily living.^20^

## Results

Table 1 reports characteristics of the study participants by fall risk category. Women in the high and low fall risk groups were not significantly different in age, ethnicity, and education. By design, because SPPB scores were used to define fall risk groups, the overall SPPB score and each of the three SPPB subscores differed significantly between groups. Average cadence during good walking was about 124 steps/min. Average values for most variables differed significantly by risk group (Table 1).

**Table 1:** Participant characteristics by fall risk category

Characteristic	Total	High fall risk (≤9 SPPB and >0 falls)	Low fall risk (>9 SPPB and 0 falls)	*p*-value
N (%)	67	19 (28.4%)	48 (71.6%)	
Age, years, mean (SD)	77.5 (6.1)	77.3 (5.9)	77.6 (6.2)	0.829
EPESE SPPB score, mean (SD)	10.1 (1.5)	8.3 (1.1)	10.8 (0.8)	<0.001
Balance subscore, mean (SD)	3.9 (0.4)	3.7 (0.7)	4.0 (0.0)	0.004
Chair stand subscore, mean (SD)	2.7 (1.1)	1.4 (0.8)	3.2 (0.8)	<0.001
Gait subscore, mean (SD)	3.5 (0.7)	3.2 (0.9)	3.6 (0.6)	0.016
Number of falls in the past year				
0 Falls	48 (71.6%)	0	48 (100%)	
1 Fall	13 (19.4%)	13 (68.4%)	0	
2–3 Falls	6 (9.0%)	6 (31.6%)	0	
Cadence (steps/minute), mean (SD)	123.5 (16.5)	120.5 (15.5)	124.6 (16.7)	<0.001
Vector magnitude CoV, mean (SD)	0.216 (0.049)	0.205 (0.047)	0.220 (0.050)	<0.001
Vector magnitude ACC, mean (SD)	0.476 (0.202)	0.500 (0.228)	0.458 (0.191)	<0.001
Vector magnitude, mean (SD)	0.998 (0.014)	0.995 (0.012)	0.998 (0.014)	<0.001
X acceleration, mean (SD)	−0.137 (0.114)	−0.103 (0.115)	−0.149 (0.112)	<0.001
Y acceleration, mean (SD)	−0.889 (0.088)	−0.910 (0.080)	−0.881 (0.089)	<0.001
Z acceleration, mean (SD)	−0.124 (0.323)	−0.044 (0.293)	−0.153 (0.328)	<0.001
X CoV, mean (SD)	−1.0 (27.8)	−1.6 (18.2)	−0.8 (30.4)	0.221
Y CoV, mean (SD)	−0.225 (0.053)	−0.206 (0.041)	−0.231 (0.055)	<0.001
Z CoV, mean (SD)	0.0 (78.9)	0.1 (153.6)	−0.1 (13.2)	0.924
X ACC, mean (SD)	0.397 (0.193)	0.392 (0.197)	0.399 (0.191)	0.109
Y ACC, mean (SD)	0.394 (0.216)	0.415 (0.239)	0.386 (0.206)	<0.001
Z ACC, mean (SD)	0.352 (0.254)	0.374 (0.270)	0.344 (0.248)	<0.001

Acceleration is measured in g’s, where 1 g = acceleration due to gravity. *p* values were calculated using two-sided Student’s *t*-tests for continuous variables and chi-square tests for categorical data

*SPPB* Short Physical Performance Battery is scored 0–12 and SPPB subscales are scored 0–4, with higher scores = better function), *CoV* coefficient of variance, *ACC* autocorrelation

The results of providing various feature sets to random forest classification models are available in Table 2. Classifiers were trained using 10-fold cross validation to ensure proper separation of training and testing data and limit overfit. The models performed with an average accuracy of 73.7%, precision (also known as positive predictive value) of 81.1%, sensitivity (also known as recall) of 84.2%, and AUC of 0.706 and could discriminate between high and low fall risk classes. The best performing feature set was feature set #10 (accuracy = 79.3%, precision = 84.6%, sensitivity = 88.1%, and AUC = 0.834), and it included: data from each axis, cross-correlations, and traditional measures of gait. Combining individual axes data into a vector magnitude (feature set #9) reduced the performance of the model dramatically (accuracy = 71.4%, precision = 78.5%, sensitivity = 84.6%, and AUC = 0.616). Traditional measures of gait alone performed the worst (accuracy = 69.0%, precision = 75.0%, precision = 87.3%, and AUC = 0.545). Models including single axis data performed better though not as well as the models including data from all three axes. Of the single axis models, the model containing vertical data outperformed mediolateral and anteroposterior models. Adding traditional measures of gait to models with signal-based features had little effect in most cases. On average, accuracy and precision differed by 0.8% and AUC differed by 0.006 compared to models containing only signal-based features. In all models containing signal-based and traditional measures of gait, signal-based measures were consistently ranked above traditional measures of gait. In the top performing models, four out of the top five features were derived from the mediolateral dimension (*z* axis) (Table 2).

**Table 2:** Performance metrics from 10-fold cross validation for random forest classification of high and low function women on each of 11 feature sets

Set	Accuracy	Precision	Sensitivity	F1-Score	AUC	Feature groups	Top-five features
1	69.0%	75.0%	0.873	0.807	0.545	Gait	STD_STEP_TIME, STD_STRIDE_TIME, MEAN_STRIDE_TIME, MEAN_STEP_TIME, CADENCE
2	71.9%	79.0%	0.845	0.817	0.665	X-axis	X_MEAN, X_COV, X_SMA, X_PFREQ, X_ENERGY
3	72.7%	79.1%	0.858	0.823	0.661	X-axis, gait	X_MEAN, X_COV, X_SMA, X_RMS, X_ENERGY
4	75.9%	82.6%	0.855	0.840	0.730	Y-axis	Y_PFREQ, Y_MCR, Y_COV, Y_STD, Y_MAD
5	76.2%	82.5%	0.862	0.843	0.727	Y-axis, gait	Y_PFREQ, Y_MCR, Y_RMS, Y_STD, Y_COV
6	70.9%	83.3%	0.760	0.795	0.759	Z-axis	Z_COV, Z_MEAN, Z_MAD, Z_STD, Z_PFREQ
7	73.1%	84.1%	0.785	0.812	0.771	Z-axis, gait	Z_COV, Z_MEAN, Z_MAD, Z_STD, Z_SMA
8	70.9%	79.3%	0.822	0.807	0.616	Vector magnitude	MAG_PFREQ, MAG_MAD, MAG_MCR, MAG_P2P, MAG_SMA
9	71.4%	78.5%	0.846	0.814	0.616	Vector magnitude, gait	MAG_PFREQ, MAG_MCR, MAG_MAD, MAG_MEAN, MAG_SMA
10	79.3%	84.6%	0.881	0.863	0.834	XYZ, cross-correlations	Z_COV, XZ_CORR, Z_MEAN, X_MEAN, Z_MAD
11	78.9%	84.4%	0.877	0.860	0.846	XYZ, cross-correlations, gait	Z_COV, XZ_CORR, Z_MEAN, X_MEAN, Z_MAD
AVG	73.7%	81.1%	0.842	0.826	0.706		

*Note*: With a two group classifier, there are 4 possible results: true positive (TP), true negative (TN), false positive (FP), and false negative (FP). Accuracy = (TP+TN)/(TP + TN + FP + FN). Precision (also referred to as positive predictive value) = TP/(TP + FP). Sensitivity (also referred to as recall) = TP/(TP + FN); F1 score = 2TP/(2TP + FP + FN); AUC = area under curve in ROC analysis

Top-five features in each feature set are listed. Full list of features included in each feature group can be found in supplementary data

*MAG* vector magnitude, *SMA* signal magnitude area, *COV* coefficient of variance, *CORR* correlation coefficient between two axes, *PFREQ* peak frequency, *MAD* mean amplitude deviation, *MCR* mean crossing rate, *STD* standard deviation, *P2P* peak-to-peak amplitude, *RMS* root mean squared. Prefixes indicate axis or vector magnitude—X anteroposterior, Y: vertical, Z: mediolateral

**Fig. 1:**
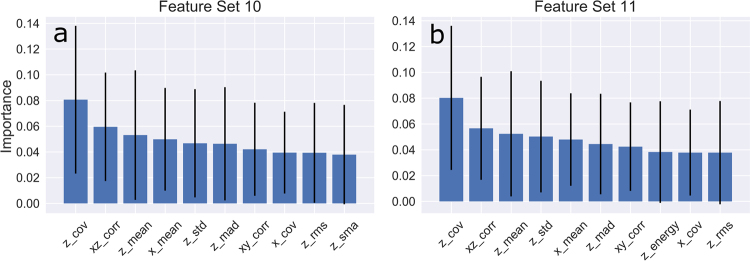
Top-ten features for two of the feature sets used in prediction of high and low function. Average importance of each feature for model prediction was computed as mean decrease impurity (see text) and is indicated by the blue bar. Black error bars represent standard deviation of importance across all trees in the forest. **a** Top-ten features for a random forest model trained on features extracted from individual *x*, *y*, and *z* axes and cross-correlations between axes. **b** Top-ten features for a random forest model trained on features extracted from the individual *x*, *y*, *z* axes, cross-correlations between axes, and traditional measures of gait

We identified the top ten features used by classifiers for feature set #10 and for feature set #11. With feature set #10 (all feature groups eligible), the most important features were mediolateral signal-based measures followed by anteroposterior signal-based measures (Fig. 1a). With feature set #11 which included traditional measures of gait, the most important features were still mediolateral and anteroposterior signal-based features (Fig. 1b). In both models, the top three features included mediolateral coefficient of variance, correlation coefficient between anteroposterior and mediolateral accelerations, and mean mediolateral acceleration. Traditional measures of gait were of lesser importance and did not rank amongst the top ten features.

## Discussion

The results suggest accelerometer-based measures of gait are potentially useful in screening older women for fall risk. Further, features derived from the accelerometer data extracted by the good walking algorithm were predictive of fall risk.^20^ Specifically, sideways (mediolateral) hip motion detected by the *z*-axis of a triaxial accelerometer may be a useful predictor of risk, such as the top three features in our analyses: coefficient of variance, correlation coefficient between two axes, and mean acceleration. The importance of features derived from *z*-axis data is plausible as excessive or variable sideways movement during walking, as measured by coefficient of variation, may increase fall risk.^21,22^ The sideways movement is consistent with age-related neuromuscular weakness due to slower motor unit recruitment with age.^23^ That is, when motor control is diminished, gait variability increases, as older adults lack the ability of younger adults to respond to perturbations in gait by increasing neuromuscular control.^24^ This may present as chaotic accelerometer tracings since erratic muscular control can cause inconsistent accelerations. The perturbation in mediolateral movement may predispose older adults to higher risk of falling sideways by exceeding the bounds of stability and may portend greater odds of a hip fracture.^25^

With the additional data, it was of little surprise that the triaxial models outperformed the single-axis models. Following the top ten features of the triaxial model, it would be expected that the mediolateral model would outperform both the anteroposterior and vertical models. However, the vertical model performed the best (accuracy = 76.2%, precision = 82.5%, and AUC = 0.727). This may be explained by certain feature pairs being more predictive than any individual feature. That is, an x-axis feature may consistently adjust the instances that end up in the next node such that the *z*-axis features are much better at separating the two classes. One possible biomechanical explanation is that vertical acceleration may be a correlate of primarily force production whereas anteroposterior and mediolateral acceleration are correlates of a combination of balance and force production. Therefore, the vertical model uses data that is more telling of an adult’s physiologic capacity to walk safely than either the anteroposterior or mediolateral models. This is supported by the significantly lower SPPB chair stand subscore of the high fall risk group (SPPB chair stand subscore = 1.4) compared to the low fall risk group (SPPB chair stand subscore = 3.2). It is difficult to assess balance in a single plane and thus requires two axes (anteroposterior and mediolateral) to completely analyze the data. Furthermore, since musculature is a component of balance, this may explain why the mediolateral and anteroposterior features are more important than vertical features in the triaxial models.

Random forest methods identified features that were reasonably predictive of fall risk, with an average accuracy of almost 73.7% and AUC of 0.706. While this level of accuracy and AUC are low compared to machine learning models that predict stable characteristics, the task of fall prediction is more challenging and the fall risk classification models met accuracy expectations. Furthermore, this average includes models that were not expected to perform well such as only traditional measures of gait and vector magnitude data. The performance of these models surpasses previous models that utilize only a single hip accelerometer during walking.^19^ Multiple features of this study could explain the increased performance such as the longer one-year falls history or combination of falls history and SPPB for fall risk classification. In-house solutions may acquire better accuracy by building higher resolution sensors or combining multiple sensors.^17,26^ Though gait quality is a strong predictor of fall risk, it is not the only risk factor. There are environmental risk factors and other host risk factors that are relatively independent of gait such as poor vision, postural hypotension, and ability of shoes to oppose slipping.

Using theoretical probabilistic models, one study estimated the maximum AUC when predicting falls within one year ranges from 0.80 to 0.89, with accuracies exceeding 80% challenging to achieve.^27^ Our models are the nearly meet these predicted maximums with an accuracy of 79.3% and AUC of 0.834 (feature set #10). Of course, it is of interest to empirically test these theoretical maximums.

The potential of signal-based accelerations as predictors of fall risk is also suggested by the fact that random forest classifiers using only signal-based features (feature set #2, #4, #6, #8, and #10) performed similarly as classifiers including traditional measures of gait (feature set #3, #5, #7, #9, and #11). This may indicate the potential of machine learning to identify interactions among signal-based features that increase their predictive ability. Reduced feature-sets have also been shown to outperform full feature-sets.^28^

The results of this study are consistent with the general finding of other studies that raw data from wearable accelerometers are potentially useful in fall prediction. Accelerometer-derived measures, including gait variability, can predict time to first fall in patients with Parkinson’s disease.^29^ The results are further consistent with other research that mediolateral and anteroposterior measures of sway and velocity are indicators of fall risk and that relatively brief gait assessments provide information on fall risk.^15^ Some studies have attained greater predictive accuracy (up to 90.4%) by using a TUG Test, rather than a simple walk test, which may better assess other risk factors including muscular strength and physiology.^30,31^ Furthermore, the models predicted fall risk based on assessments rather than actual falls history.^31^ When using past falls history, reasonable accuracy, sensitivity, and specificity (80%, 74%, 96%, respectively) was achieved using accelerometer data from only a TUG test and a 20 m walk.^32^ However, these studies utilized a homebrew accelerometer solution which may contain better sensors than commercial offerings but require expertise to implement.^30–32^ Greater accuracy, sensitivity, and specificity can be achieved with multiple sensors on body parts other than the waist.^26^ This finding suggests use of accelerometer to assess characteristics of movement beyond only gait characteristics may improve predictive ability.

Of course, this pilot study has several limitations. First, the study assumes that gait was stable between the measurement of SPPB in 2012–13 and data collection of the calibration substudy up to many months later. Second, as in other laboratory studies of gait and fall risk, women may alter their gait in laboratory conditions under investigator observation.^33^ Third, the study has a small sample size with uneven numbers of women in the two risk groups. It did not attempt to classify fall risk in all women but only in women at upper and lower ends of risk. Fourth, the study used data on past falls rather than prospectively collected information after the calibration study. Possibly, gait characteristics at the time of a past fall could differ from gait characteristics at the time of the 400 m walk, e.g., a participant began walking more cautiously after a fall. Fifth, machine learning methods are susceptible to overfitting prediction models, though the cross-validation method of this study, combined with both the tree bagging and feature bagging used in random forests, is less likely to have overfit than the base method of using a single decision tree. Finally, because women were screened for ability to walk on a treadmill, the sample excluded women at highest fall risk for whom treadmill walking is unsafe. For example, the sample did not include any women with 4+ falls in the past year. In a study where the “high risk” group includes frequent fallers, classification accuracy might be improved and, possibly, different features or additional features could be included in predictive models.

## Conclusion

This pilot study suggests that raw data collected from a hip-worn, triaxial accelerometer during walking may be useful in assessing risk of falls. Prospective studies of the ability of accelerometer-based measures of walking to predict falls are warranted, given the potential of these inexpensive sensors to monitor walking and fall risk during activities of daily life, in large numbers of older adults, and over long periods of time. In particular, large prospective studies in older adults who vary widely in risk of falls are needed. In these studies, accelerometer-based assessments of fall risk should be based upon patterns of walking under free-living conditions, rather than only on data collected in laboratory or clinical settings. Analysis of data collected in free-living conditions may identify different gait characteristics as indicators of fall risk, in part because free-living walking occurs in a variety of environments (e.g., hills, uneven sidewalks, and wet surfaces).

We are proceeding to confirm the usefulness of accelerometer-based measures of walking in predicting fall risk using an OPACH dataset of over 5000 women who wore a hip accelerometer for 1 week and reported falls prospectively for 1 year. With this unique dataset, we seek to analyze which of the accelerometer-derived features identified in the current study are useful in predicting fall risk in real-world free-living conditions. We also seek to determine if these features improve ability to predict fall risk compared to standard prediction models based upon known fall risk factors and physical function measures.

If accelerometer-based, fall risk models based upon prospective data are successful, then risk screening with smartphones could become feasible for large populations, by simply carrying these during normal activities as passive monitors. In separate preliminary work, accelerometer data extracted from smartphones during week-long activities of daily living was input to predictive models of pulmonary function and accurately predicted pulmonary function in senior patients with nearly 100% accuracy.^20^ The least expensive low-end smartphones (e.g., the LG Optimus Zone 3 which now costs $30) can measure gait as accurately as the most expensive high-end medical accelerometers, while also being more accurate than fitness devices.^34^ Thus, there is a potential path towards screening and prevention of falls at population scale for the aging population, by leveraging already carried personal phones.

## Methods

### Study population

Participants in the current study were 67 women originally recruited for the Women’s Health Initiative (WHI) in the 1990s who subsequently consented to participate in the second extension study of WHI (2010–2015) and in a WHI ancillary study called OPACH (Objective Physical Activity and Cardiovascular Health in Older Women, R01 HL105065; PI: A LaCroix). The 67 women further consented to participate in a substudy which calibrated the accelerometers used in OPACH. The methods of both OPACH and the calibration substudy are described elsewhere.^35,36^ Thus this study is a secondary analysis of OPACH data for the purpose of exploring predictive models of fall risk.

In brief, the subset of OPACH participants included in the present study had an in-home visit for data collection and completed a physical activity questionnaire between March 2012 and May 2013. In 2013, they were recruited to participate in the calibration study. Notably, an exclusion criterion for the calibration substudy was a significant change in health status affecting ability to walk or risk of walking-related injury between OPACH data collection and recruitment for the substudy. Of the *N* = 7058 women enrolled in OPACH, *N* = 142 participated in the calibration substudy, and *N* = 67 of these women met the inclusion criteria for this study: (1) completed the SPPB at the in-home visit (scored 0–12 with higher scores indicate better function); (2) provided data on history of falls in the past year on the OPACH Physical Activity Questionnaire; (3) completed the 400-m walk of the calibration study; and (4) met criteria for either high fall risk or low fall risk. Consent to participate in OPACH was obtained by either phone or mail. After a screening phone interview, participants in the calibration substudy provided written consent at the study’s clinic visit. The OPACH study and the calibration substudy were approved by the Institutional Review Boards at each clinical site and by the WHI Clinical Coordinating Center.

#### Classification of high and low fall risk

We decided to classify fall risk of calibration study participants based upon two, well-known predictors of fall risk: history of falls in the past year and SPPB score. The CDC also uses these predictors to classify fall risk in the STEADI Toolkit.^3^ In a study of 66,134 postmenopausal women, the strongest predictor of future falls was any fall in the past 12 months.^37^ A study of the SPPB and fall risk concluded that, in older women, SPPB scores of 9 or less are associated with higher fall risk in women.^4^ Hence, we classified women as “high fall risk” (*N* = 19) based on SPPB scores of ≤9 and reporting of at least one fall in the past year. We classified women as “low fall risk” (*N* = 47) based on SPPB score of 10–12 and no reported falls in the past year. For the purposes of this pilot study which has limited statistical power, we decided not to attempt a more difficult classification task of distinguishing between women of high fall risk versus intermediate fall risk (i.e., women with past falls but SPPB scores of 10–12, or women with SPPB scores of 9 or less, but no past falls).

### Study measures

#### OPACH and the WHI extension study

Demographic data (age, ethnicity, education) were available as part of the 2nd WHI extension study. The SPPB was collected at the in-home visit. The SPPB includes a chair stand test (score 0–4), a balance test (score 0–4), and a gait speed test (scored 0–4), with total score ranging from 0–12 (higher scores indicate better function). Participants self-reported the number of falls in the past year on the OPACH Physical Activity Questionnaire (available in an online supplement to the Design paper).^35^

#### Calibration substudy

Height and weight were measured at the calibration substudy clinic visit. Women further completed up to eight tasks including a 400-m walk test during the calibration study visit. The 400 m walk test involved 10 laps around an indoor hallway course marked by cones 20 m apart. Women walked without aids at a normal pace. During all tasks, women wore a triaxial accelerometer (ActiGraph GT3X+; Pensacola, Florida), a POLAR heart rate monitor, and an Oxycon—a portable, battery-operated metabolic unit (Oxycon Mobile; CareFusion, Rolle, Switzerland). The accelerometer was set to collect raw data at 30 Hz. Raw data were downloaded from the device using the manufacturer’s ActiLife software. Vector magnitude for each 1/30th of a second was computed from raw data as the square root of the sum of the *x*, *y*, and *z* axis accelerations squared.

### Predictive modeling

#### Preprocessing

During the 400 m walk test, women needed to turn 180 degrees at the cones, and some women would occasionally pause during the test. The purpose of preprocessing was to separate data on steady or “good” walking from data on turns and pauses. In more detail, “good walking” refers to a steady pattern of walking like that seen during a straight path walk test. Previous research effectively utilized this algorithm as part of a pipeline for gait analysis and highly accurate prediction of pulmonary function in both laboratory and free-living environments.^38,39^ The patient population was similar in age to the individuals in this study, although it may be important to note that the individuals from the previous work were patients undergoing pulmonary rehabilitation in a suburban hospital.^38,39^

First, accelerometer tracings for each participant were segmented using a ten-second sliding window with 50% overlap. A variety of window sizes have been used to segment time series for classification tasks and often range from one to ten seconds in length with a median length of three seconds.^40^ We selected a window size of ten seconds to ensure the capture of a sufficient number of steps necessary for computation of traditional measures of gait such as those that look at variation in step and stride. It should be noted that the use of “large” windows leads to increased computation time for all features and inherently imposes a limit on the resolution at which *changes* in activity can be identified.^41^ Regarding signal-based features, larger windows have been shown to provide minimal increase in accuracy compared to windows of 1–3.25 s.^41^ For this calibration study, these limitations are less of a concern given that all subjects are either walking or not walking during the test (few activity transitions) and the 400 m distance allows for the collection of plenty of data. For free-living data, signal segmentation becomes quite important given the increased variation in both task type and duration. Second, segments (periods of time) containing non-walking elements were eliminated through two filtering steps. For segment filtering, the vector magnitude was computed and subjected to a threshold-based filter which discarded segments of low standard deviation (VM standard deviation < 0.01) to remove periods of inactivity. This threshold was experimentally determined through manual inspection of the accelerometer tracings from a randomized subset of individuals and fixed to ensure that all walking segments were retained. This threshold value was not unique for each participant but instead fixed for all participants analyzed in this study.

**Fig. 2:**
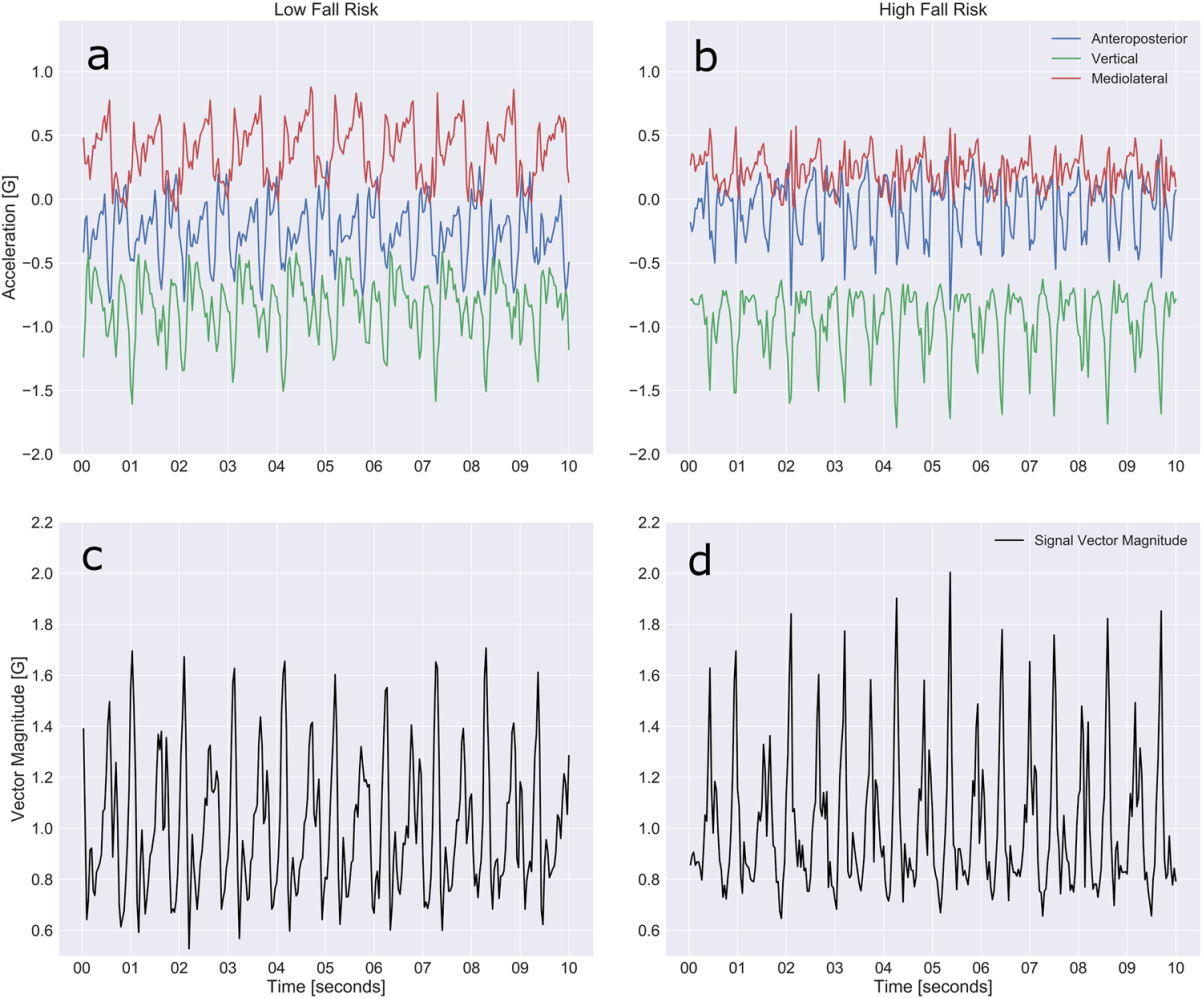
Ten second acceleration tracings from the 400-m walk for a participant classified as high fall risk woman (**a**, **c**) and a participant classified as low fall risk (**b**, **d**). **a**, **b** shows acceleration data in all three accelerometer axes: anteroposterior (blue), vertical (green), and mediolateral (red). **c**, **d** shows vector magnitude of acceleration for both participants

**Fig. 3:**
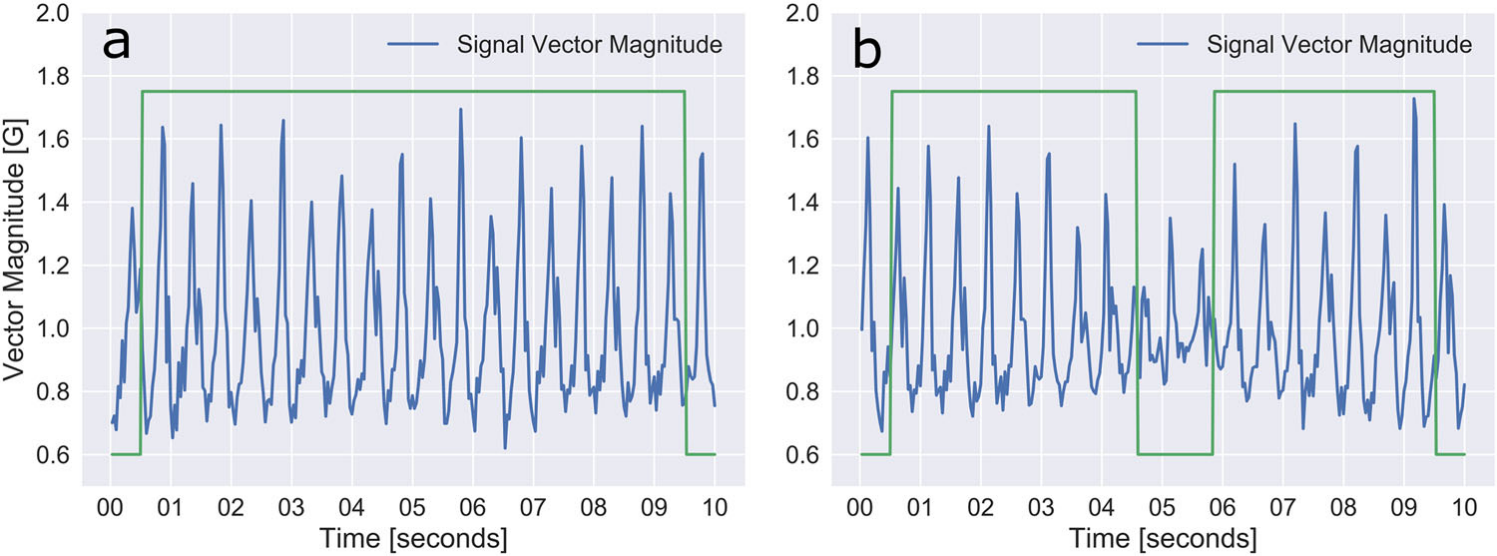
Example accelerometer vector magnitude from a single individual during the 6MWT. **a** A ten-second period of smooth walking without any turns. The “good walking” algorithm identifies the full segment as good walking (high green line). **b** A ten-second period of smooth walking which contains a turn. The “good walking” algorithm identifies the substantial reduction in acceleration magnitude and eliminates this portion of the walk tracing (green line drops down). Note, the first and last 15 data points in any segment are always identified as “non-walking” due to insufficient information

Third, periods during walking disturbed by turns and pauses were excluded from analysis using our algorithm previously developed to identify “good walking”.^20^ As previously mentioned, good walking identified with this approach was used as input to predictive models for pulmonary function, and these models showed high accuracy.^38,39^ Figure 2 contains a sample of two 10-s periods of vector magnitude calculated from accelerometer raw data. The algorithm identifies repetitive patterns of “good walking” and eliminates portions of the tracing without the good walking pattern (Fig. 3).

#### Feature engineering

Features were extracted from the remaining ten-second good walking samples. Signal-based features in the time and frequency domains were computed for each of the individual accelerometer axes and the vector magnitude. The features selected for extraction were chosen based upon both knowledge of how gait affects fall risk and upon findings of research on assessing fall risk using inertial, wearable sensors.^15^ Features were organized into groups to assess the relative predictive ability of traditional measures of gait and signal-based features of the accelerometer data. The specific features in each feature group can be found in Table 3.

**Table 3:** Full list of features used for model training

Category	Features
Signal-based features	Mean
	Standard deviation (STD)
	Root mean squared (RMS)
	Autocorrelation coefficient (ACC)
	Coefficient of variance (COV)
	Mean crossing rate (MCR)
	Signal magnitude area (SMA)
	Peak-to-peak amplitude (P2P)
	Peak frequency (PFREQ)
	Energy
	Mean amplitude deviation (MAD)
	XY cross-correlation (XY_CORR)
	YZ cross-correlation (YZ_CORR)
	XZ cross-correlation (XZ_CORR)
Traditional measures of gait	Cadence [steps/minute]
	Stride time [seconds](mean and standard deviation)
	Step time [seconds] (mean and standard deviation)

#### Selection of optimal model for use in feature selection

Certain machine learning models can be used to identify a subset of features that capture the most useful content for a larger classification problem—a process called “feature selection.” Some models available for this purpose include Decision Trees, Random Forests, and Support Vector Machines. To determine the model likely best suited for this study’s classification task, a simple spot-checking approach was used. This approach involved training each classifier (model) with default parameters on the full feature set and evaluating performance via 10-fold cross validation. With 10-fold cross validation, the data were divided into ten equally-sized partitions with nine partitions used for model training and one for testing. This process was repeated such that each partition was used once for testing. Performance metrics averaged across all ten folds were used to compare model performance and included accuracy, precision (positive predictive value), recall (true positive rate or sensitivity), F1-score (harmonic mean of precision and recall), and area under the ROC curve (AUC). Based upon the results, it was deemed that Random Forests were likely the most appropriate classifier to use for the study task.

#### Random forest training and evaluation

Random forests of 500 trees with a maximum depth of ten nodes were trained and evaluated using 10-fold cross validation implemented in the scikit-learn library.^42^ Again, metrics including accuracy, precision, recall, F1-score, and AUC were used to assess performance. Separate forests were trained on each of 11 feature sets to obtain further insight into the usefulness of certain feature types and the combined effects of certain feature groups.

#### Feature importance

Relative feature importance in random forests was characterized by mean decrease impurity.^43^ Impurity is computed by summing the weighted reduction of sample entropy for all splits that utilize the feature of interest. The resulting values are then averaged across all trees in the forest. Feature importance was calculated independently for forests trained on each of the seven feature sets. The top-ten features were identified for each forest and used to assess feature applicability to fall risk prediction.

### Data availability

The data that support the findings of this study are available on request from Andrea Z. Lacroix (A.Z.L., alacroix@ucsd.edu). The data are not publicly available now due to the ongoing main WHI study. Data will be made available after the main study results have been published. Data can be accessed through WHI data sharing policies described at https://www.whi.org/researchers/data/Pages/Home.aspx.
